# HA-Coated PLGA Nanoparticles Loaded with Apigenin for Colon Cancer with High Expression of CD44

**DOI:** 10.3390/molecules28227565

**Published:** 2023-11-13

**Authors:** Bo Yang, Yongqing Mao, Yanjun Zhang, Yue Hao, Meitong Guo, Bian Li, Haisheng Peng

**Affiliations:** 1 School of Pharmacy, Harbin University of Commerce, Harbin 150076, China; 2 Department of Pharmacology, Medical College, University of Shaoxing, Shaoxing 312000, China; 3 Heilongjiang Provincial Key Laboratory of Neurobiology, Department of Neurobiology, Harbin Medical University, Harbin 150086, China

**Keywords:** API, PLGA nanoparticles, HA, CD44, colon cancer, targeted delivery

## Abstract

Apigenin (API) possesses excellent antitumor properties but its limited water solubility and low bioavailability restrict its therapeutic impact. Thus, a suitable delivery system is needed to overcome these limitations and improve the therapeutic efficiency. Poly (lactic-co-glycolic acid) (PLGA) is a copolymer extensively utilized in drug delivery. Hyaluronic acid (HA) is a major extracellular matrix component and can specifically bind to CD44 on colon cancer cells. Herein, we aimed to prepare receptor-selective HA-coated PLGA nanoparticles (HA-PLGA-API-NPs) for colon cancers with high expression of CD44; chitosan (CS) was introduced into the system as an intermediate, simultaneously binding HA and PLGA through electrostatic interaction to facilitate a tighter connection between them. API was encapsulated in PLGA to obtain PLGA-API-NPs, which were then sequentially coated with CS and HA to form HA-PLGA-API-NPs. HA-PLGA-API-NPs had a stronger sustained-release capability. The cellular uptake of HA-PLGA-API-NPs was enhanced in HT-29 cells with high expression of CD44. In vivo, HA-PLGA-API-NPs showed enhanced targeting specificity towards the HT-29 ectopic tumor model in nude mice in comparison with PLGA-API-NPs. Overall, HA-PLGA-API-NPs were an effective drug delivery platform for API in the treatment of colon cancers with high expression of CD44.

## 1. Introduction

Apigenin (API) is a natural flavone that is abundant in a range of foods and medicinal herbs, such as oranges, celery, and chamomile [1,2]. Like other flavonoids, API possesses antioxidant, anti-inflammatory, and anti-cancer properties, among which, the anti-cancer properties of API have attracted particular attention [3,4]. API has been demonstrated to inhibit various cancers through multiple cellular mechanisms, including preventing cell invasion and migration, promoting the immune response, and triggering cell apoptosis and autophagy. In vitro and in vivo studies have also revealed that API could inhibit tumor migration [4,5,6,7]. However, although API holds promising therapeutic potential, its limited water solubility and low bioavailability restrict its ability to effectively reach target tissues, reducing its therapeutic impact [8]. Therefore, there is an urgent need for developing suitable delivery systems to enhance the solubility and bioavailability of API for the improvement of the therapeutic outcomes.

Poly (lactic-co-glycolic acid) (PLGA) is a copolymer that has been extensively studied and utilized in the biomedical field due to its perfect biocompatibility and biodegradability [9]. PLGA nanoparticles (NPs) are submicron colloids that can encapsulate various therapeutic agents, from small molecules to larger biomolecules such as proteins or RNA [10,11]. Compared with other drug delivery systems, PLGA NPs offer several advantages, including perfect biodegradability and biocompatibility, adjustable degradation rates, and the excellent capacity to encapsulate a variety of drugs [12,13]. Thus, PLGA NPs have emerged as a highly popular and widely adopted technology in the field of drug delivery.

Hyaluronic acid (HA) is a major extracellular matrix component that is characterized as a non-sulfated, linear glycosaminoglycan composed of repeating disaccharide moieties of d-glucuronic acid and N-acetyl-D-glucosamine [14]. Previous studies have shown that HA can specifically bind with the CD44 receptors [15,16], which are notably overexpressed in colon cancer cells and can promote progression and metastasis [17]. The interaction between HA and CD44 offers possibilities for targeted cancer therapy in colon cancer. Zhu C et al. engineered smart nanomicelles by conjugating HA totocopherol succinic acid via disulfide bonds, facilitating the precision targeting of paclitaxel delivery to the CD44 receptors. This mechanism markedly inhibited the proliferation and metastatic tendencies of colon cancer cells in situ, enhancing therapeutic efficacy while reducing side effects [18,19]. Therefore, HA-modified nanoparticles can be used for targeted delivery to CD44 overexpressing colon cancer cells. However, both PLGA NPs and HA carry surface positive charges. To achieve the encapsulation of HA on the surface of PLGA NPs, we introduced chitosan (CS) derived from crustacean exoskeletons. CS carries a negative charge on its surface, allowing it to initially bind to PLGA NPs through electrostatic adsorption and subsequently further bind to HA. This electrostatic interaction played a crucial bridging role, enabling the successful encapsulation of HA on the outer layer of PLGA NPs [20].

Hence, in this study, we aimed to prepare a receptor-selective HA-coated PLGA nanoparticle drug delivery system (HA-PLGA-API-NPs) to achieve the targeted delivery of API to colon cancer cells that overexpress CD44. The particle size and zeta potential of all PLGA NPs were characterized using dynamic light scattering (DLS). The encapsulation efficiency (EE), drug loading (DL), and in vitro release kinetics of HA-PLGA-API-NPs were determined using high-performance liquid chromatography (HPLC) and ultrafiltration centrifugation methods. Cell uptake experiments and small animal in vivo imaging techniques were employed to investigate the targeting ability of HA-PLGA-API-NPs in vitro and in vivo. The results revealed that HA-PLGA-API-NPs possessed a uniform size and good stability. HA-PLGA-API-NPs showed improved sustained-release capabilities compared with that of PLGA-API-NPs. Cellular uptake of HA-PLGA-API-NPs was enhanced in HT-29 cells with high expression of CD44. HA-PLGA-API-NPs showed enhanced targeting specificity towards the HT-29 ectopic tumor model in nude mice in comparison with PLGA-API-NPs.

## 2. Results

### 2.1. Preparation and Characterization of HA-Coated PLGA-API-NPs

**Figure 1:**
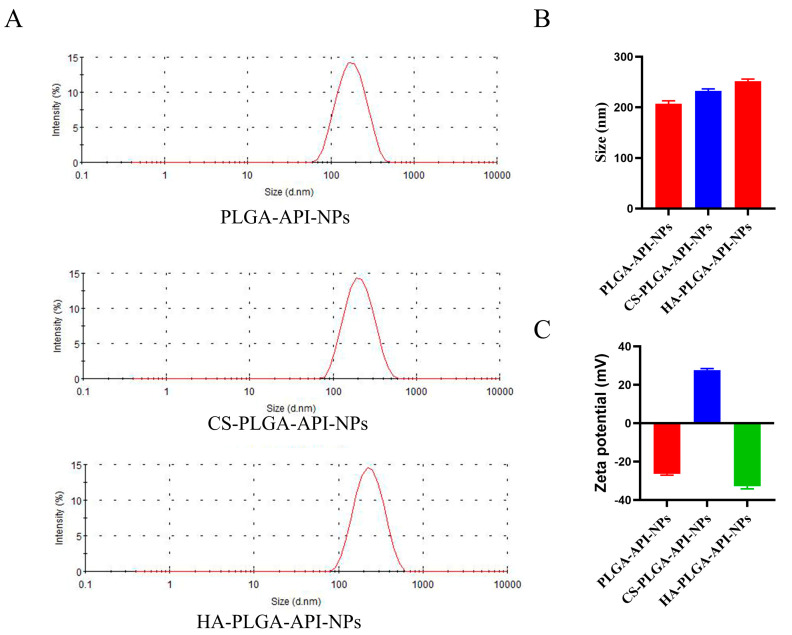
The size distributions (**A**,**B**) and zeta potentials (**C**) of PLGA-API-NPs, CS-PLGA-API-NPs, and HA-PLGA-API-NPs. The data are presented as means ± SD, *n* = 3.

As shown in Figure 1A,B, the average sizes of PLGA-API-NPs, CS-PLGA-API-NPs, and HA-PLGA-API-NPs were 210.3 nm, 234.3 nm, and 247.2 nm, respectively. The particle sizes of the CS-PLGA-API-NPs were enhanced by about 13 nm after the HA coating. PLGA-API-NPs, CS-PLGA-API-NPs, and HA-PLGA-API-NPs had zeta potentials of −26.3 mV, +27.9 mV, and −32.7 mV (Figure 1C), respectively, indicating the CS coating of the PLGA nanoparticles followed by HA. The EE and DL values for API in the HA-PLGA-API-NPs were (90.56 ± 0.57) % and (3.19 ± 0.03) %, respectively. The stability assessment results are presented in Table 1 and show a slight increase in nanoparticle size over 10 days in 4 °C conditions, within a range of approximately 10 nm, and an increase of around 20 nm after 10 days at 25 °C. The DL of API had decreased by about 0.4% within 10 days at 4 °C and by approximately 0.6% over a 10-day period at 25 °C. Although slight variations in the particle size and drug loading of API were presented under both 4 °C and 25 °C conditions, these changes remained within an acceptable range, indicating that the HA-PLGA-API-NPs in this study can be stably stored for approximately 10 days. However, extending the observation period for nanoparticles is necessary to conduct a more comprehensive stability assessment in future research.

### 2.2. Cumulative Release Rate In Vitro

**Figure 2:**
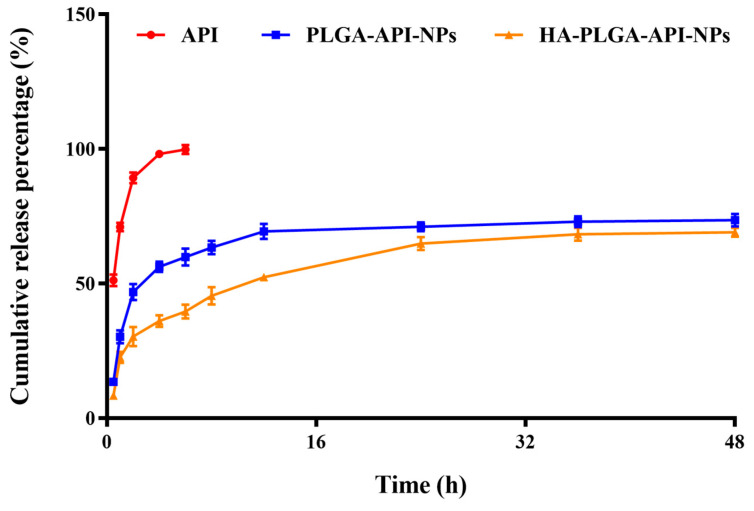
The release of API from API, PLGA-API-NPs, and HA-PLGA-API-NPs in release media (pH 7.4 phosphate buffer containing 20% ethanol). The data are presented as means ± SD (*n* = 3).

The in vitro cumulative release rates of free API, CS-PLGA-API-NPs and HA-PLGA-API-NPs were calculated according to Formula (II). As shown in Figure 2, the uncoated PLGA-API-NPs released over 70% of the API within 24 h, whereas HA-PLGA-API-NPs required 48 h to release 68% of the API. The release curve results indicated that uncoated PLGA-API-NPs exhibited rapid release, whereas HA-PLGA-API-NPs displayed sustained and controlled release over an extended period. This suggested that the HA coating acted as a barrier, effectively modulating and controlling the release of API. Such a controlled release may offer therapeutic advantages when prolonged drug release is desired.

### 2.3. In Vitro Cellular Uptake Assay

**Figure 3:**
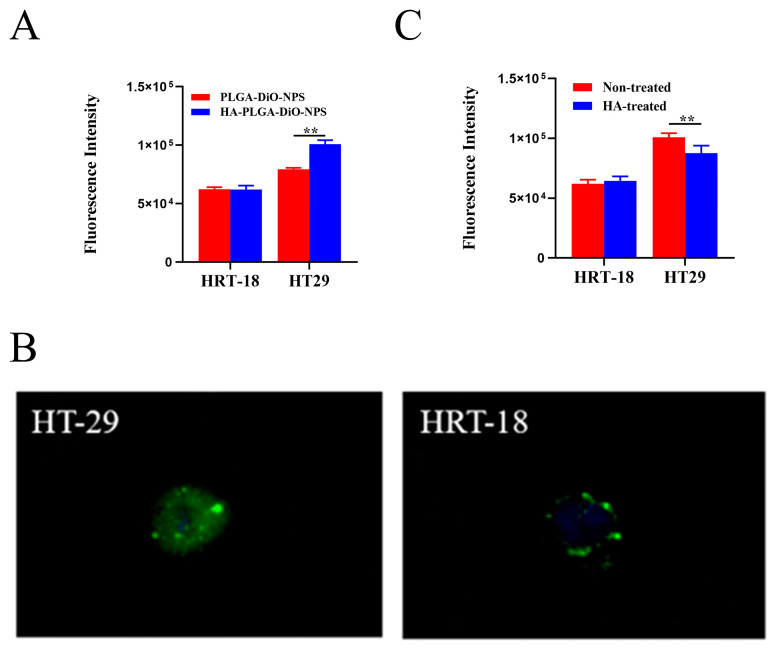
Cell uptake. (**A**) Uptake of PLGA-DiO-NPs and HA-PLGA-DiO-NPs by HT-29 and HRT-18 cells. (**B**) Fluorescence pictures of HA-PLGA-DiO-NP uptake by HT-29 and HRT-18 cells. (**C**) Uptake of HA-PLGA-DiO-NPs by HT-29 and HRT-18 cells with and without HA treatment. All data are presented as means ± SD (*n* = 3), ** *p* < 0.01.

Previous studies have shown that the CD44 receptor expression in HT-29 cells is significantly higher than in HRT-18 cells [21,22,23]. Therefore, in the subsequent experiments, HT-29 cells and HRT-18 cells were used as models for high and low CD44 expression, respectively. To assess CD44-receptor-mediated cellular internalization, HT-29 cells and HRT-18 cells were incubated with PLGA-DiO-NPs or HA-PLGA-DiO-NPs for 4 h. As shown in Figure 3A, HT-29 cells exhibited a significantly higher uptake of DiO compared with HRT-18 cells. Furthermore, the uptake of HA-PLGA-DiO-NPs by HT-29 cells was enhanced in comparison with the uptake of PLGA-DiO-NPs after HA encapsulation. Figure 3B revealed that HT-29 cells exhibited stronger green fluorescence after co-incubation with HA-PLGA-DiO-NPs, whereas HRT-18 cells displayed minimal green fluorescence. This suggested that HT-29 cells took up more HA-PLGA-DiO-NPs than HRT-18 cells did, and this was most likely due to the HA binding to CD44 on the surface of HT-29 cells and promoting the uptake of HA-PLGA-DiO-NPs by HT-29 cells. In order to further validate the role of HA in nanoparticle cellular uptake, we pre-treated HT-29 cells and HRT-18 cells with free HA. Figure 3C showed that the uptake of HA-PLGA-DiO-NPs by HT-29 cells significantly decreased after a 4 h pre-incubation with free HA, whereas there was no significant change in the uptake of HA-PLGA-DiO-NPs by HRT-18 cells following a 4 h pre-incubation with free HA. All these results confirmed that HT-29 cells with high CD44 expression exhibited a greater uptake ability for HA-PLGA-DiO-NPs compared with HRT-18 cells with low CD44 expression. The HA coating might be the primary reason for the enhanced uptake of HA-PLGA-DiO-NPs by HT-29 cells and the targeting capability of PLGA-DiO-NPs for HT-29 cells in vitro.

### 2.4. In Vitro Cytotoxicity of HA-PLGA-API-NPs

**Figure 4:**
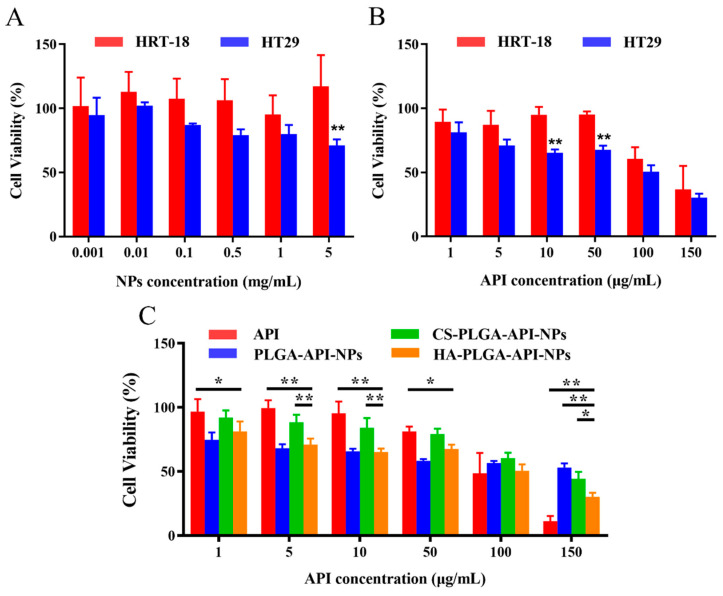
Cells and cytotoxicity (*n* = 6). (**A**) Cytotoxicity assay using HT-29 and HRT-18 cells treated with blank nanoparticles. (**B**) Cytotoxicity assay using HT-29 and HRT-18 cells treated with HA-PLGA-API-NPs. (**C**) Cytotoxicity of free API and different modified nanoparticles towards HT-29 cells. The data are presented as means ± SD, *n* = 3, * *p* < 0.05, ** *p* < 0.01.

The capability of a drug to induce cytotoxicity in cancer cells frequently serves as a benchmark for its anticancer potential, with greater cytotoxicity conferring stronger anticancer activity. As shown in Figure 4A, the viability of HRT-18 cells remained above 80% after incubation with different concentrations of HA-PLGA-NPs, which indicated that the empty nanoparticle vector had good biological safety. However, the viability of HT-29 cells remained above 80% when the concentration of HA-PLGA-NPs was less than 0.1 mg/L but significantly decreased to around 65% when the concentration of HA-PLGA-NPs reached 5 mg/L. This was probably because of the presence of the substantial number of CD44 receptors on HT-29 cells resulting in a higher uptake of NPs compared with the HRT-18 cells. Although HA-PLGA-NPs have relatively low toxicity, an excessive quantity of NPs taken up by cells could transport significant amounts of various excipients into the cell, thereby disrupting the nutrient uptake of the cells and potentially resulting in cell death. Next, different concentrations of HA-PLGA-API-NPs and HRT-18 and HT-29 cells were incubated for 24 h. The MTT results are shown in Figure 4B. The survival rate of HRT-18 cells decreased to 60.55% when the concentration of API reached 100 μg/mL, whereas that of HT-29 cells decreased significantly to 62.36% when the concentration of API reached 10 μg/mL, which indicated that HA-PLGA-API-NPs had a stronger growth inhibition effect on HT-29 cells with high CD44 overexpression. To further illustrate the cytotoxicity of HA-PLGA-API-NPs towards HT-29 cells, different concentrations of free API, PLGA-API-NPs, CS-PLGA-API-NPs, and HA-PLGA-API-NPs were incubated with HT-29 cells. Figure 4C indicates that the cytotoxicity of PLGA-API-NPs towards HT-29 cells was sequentially greater than that of HA-PLGA-API-NPs, free API, and CS-PLGA-API-NPs at API concentrations below 50 μg/mL. This could be attributed to the facts that the uptake of PLGA-API-NPs was greater than that of free apigenin and the release of API from PLGA-API-NPs was higher than that from CS-PLGA-API-NPs and HA-PLGA-API-NPs at lower API concentrations, thus resulting in the highest cell toxicity. Compared with CS-PLGA-API-NPs, the increased cytotoxicity of HA-PLGA-API-NPs might primarily be attributed to the enhanced cellular uptake of HA-PLGA-API-NPs after the HA coating. As the concentration increased, the sequence of cytotoxicity towards HT-29 cells shifted from free API, HA-PLGA-API-NPs, and CS-PLGA-API-NPs to PLGA-API-NPs. This might be because the influence of cellular uptake on cytotoxicity gradually surpassed the impact of drug release with the increasing nanoparticle concentration. Therefore, the cytotoxicity of HA-PLGA-API-NPs was sequentially greater than that of CS-PLGA-API-NPs and PLGA-API-NPs. However, there was limited release of apigenin from the nanoparticles within 24 h, resulting in the highest cellular toxicity being exhibited by free API.

### 2.5. In Vivo NIRF Imaging

**Figure 5:**
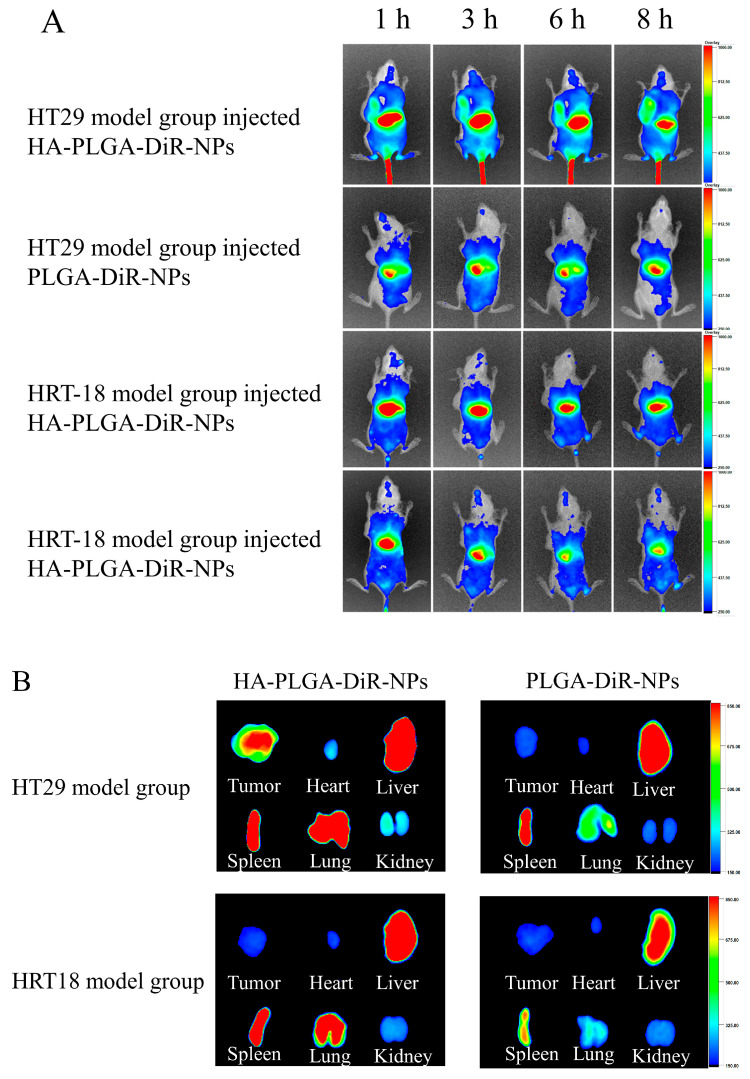
CD44-mediated tumor targeting delivery of HA-PLGA-DiR-NPs. (**A**) In vivo fluorescent small animal imaging of two model rats injected with PLGA-DiR-NPs or HA-PLGA-DiR-NPs. (**B**) Visceral fluorescence images of two model rats after injection of PLGA-DiR-NPs or HA-PLGA-DiR-NPs. The data are presented as means ± SD (*n* = 3).

In order to predict the antitumor efficacy of nanoparticles, biodistribution studies on HA-PLGA-DiR-NPs were conducted to investigate their in vivo targeting behavior. PLGA-DiR-NPs and HA-PLGA-DiR-NPs were injected intravenously into mice bearing one of the two tumors; the mice were then monitored using near-infrared optical imaging. Figure 5A shows the fluorescent images of mice at 1, 3, 6, and 8 h postinjection. Compared with the PLGA-DiR-NP mice group, stronger fluorescence signals were observed at the HT-29 tumor sites of HA-PLGA-DiR-NP group throughout the experiment, whereas both formulations showed less distribution in HRT-18 model mice. These results demonstrated greater accumulation of HA-PLGA-DiR-NPs at the HT-29 tumor site due to an HA-mediated active targeting effect, since HRT-18 cells expressed fewer CD44 receptors and DiR. The fluorescence intensity at the tumor sites of both groups peaked at 8 h postinjection and then decreased gradually, indicating this was the optimal laser irradiation timepoint. However, tumor fluorescence intensity was significantly stronger in the HA-PLGA-DiR-NP group than in the PLGA-DiR-NP group at 8 h postinjection, indicating the satisfactory targeting ability of the HA-PLGA-DiR-NPs. The excised organs and tumors were measured for fluorescence intensity at 8 h postinjection (Figure 5B). Tumors from the HA-PLGA-DiR-NP mice group showed significantly stronger fluorescence than those from the PLGA-DiR-NP group. Notably, the kidneys and hearts of both groups displayed only weak fluorescence, suggesting minimal side effects. These results demonstrated that HA-PLGA-DiR-NPs could efficiently accumulate into tumor sites via an HA-mediated active targeting effect, maximizing the antitumor effects and minimizing side effects.

## 3. Materials and Methods

### 3.1. Materials

Hyaluronic acid (Molecular weight 50~80 kDa) and chitosan were obtained from BASF Biotechnology Co., Ltd. (Zibo, China). PLGA (100 kDa) was from Dai Gang Biological Technology Co., Ltd. (Jinan, China). API was obtained from Yuquan Biological Technology Co., Ltd. (Taiyuan, China). Tracking probes including 3,3-dioctadecyloxacarbocyanine perchlorate (DiO), 4′,6-Diamidino-2′-phenylindole (DAPI), and 1,1-dioctadecyl-3,3,3,3-tetramethylindotricarbocyanine iodide (DiR) were purchased from Beijing Fanbo Science & Technology Co., Ltd. (Beijing, China). Anhydrous ethanol, dichloromethane, the surfactant F68, and Tween 80 were purchased from National Pharmaceutical Group Chemical Reagent Co., Ltd. (Beijing, China). The colon cancer cell lines HT-29 and HRT-18 were obtained from the Shanghai Institute of the Chinese Academy of Sciences (Shanghai, China).

### 3.2. Preparation and Characterization of PLGA Nanoparticles

According to the prescription screening and a previous publication [24], API (6 mg) and PLGA (60 mg) were accurately weighed and dissolved into a mixture of ethanol (3 mL) and dichloromethane (4.5 mL) as the oil phase. Briefly, the surfactant F68 (180 mg), chitosan (4.5 mg), and Tween 80 (0.225 g) were added to 45 mL of ultrapure water as the aqueous phase. All lipid materials were completely dissolved using a magnetic agitator to form a clear and transparent solution. The oil phase was slowly added to the aqueous phase and stirred for 10 min to form an emulsion. The oil phase was slowly added to the aqueous phase and stirred for 10 min to form an emulsion. Subsequently, the emulsion was added to 100 mL of ultrapure water and homogenized using a high-pressure homogenizer, followed by stirring for 12 h. CS-PLGA-API-NPs were then obtained by centrifuging the mixture for 45 min at 20,000× *g* at room temperature. The obtained nanoparticles were immediately resuspended in 30 mL of acetic acid buffer solution (4 °C, pH 6.8) containing HA (0.25 mg/mL). The mixture was then stirred for 1 h and filtered through a 0.45 μm polycarbonate ester membrane to obtain the HA-PLGA-API-NPs, which was lyophilized and stored. DiO and DiR are both lipophilic dyes used for cellular and tissue labeling in biological research. Fluorescence labeling was achieved by substituting API with DiO or DiR to prepare DiO- or DiR-labeled HA-PLGA-API-NPs. The size distribution and zeta potential of PLGA-API-NPs, CS-PLGA-API-NPs, and HA-PLGA-API-NPs were measured via dynamic light scattering at room temperature (Malven Zetasizer Nano ZS90, Malvern Panalytical, Malvern, UK).

### 3.3. Drug Loading Amount

The drug loading (DL) amount and encapsulation efficiency (EE) of the PLGA-API-NPs were determined via high-performance liquid chromatography (HPLC) after ultrafiltration centrifugation. Briefly, newly prepared PLGA-API-NPs were lyophilized and the weight of nanoparticles (*W_n_*) was recorded. PLGA-API-NPs were then subsequently added into a 10 mL brown volumetric flask. Demulsification was carried out using 1 mL methanol, followed by ultrasonic dispersion for 2 min and further mixing through vortexing and shaking for 3 min. Next, the mixture was made up to 10 mL with a liquid (mobile phase) consisting of acetonitrile and water at a *v*/*v* ratio of 41:59, followed by ultrasonic dispersion for 30 min and filtering through a 0.22 μm microporous filter membrane. The total amount of API in the PLGA-API-NPs (*W_t_*) was tested via HPLC. At the same time, the same quantity of PLGA-API-NPs was weighed and added to an ultrafiltration centrifuge tube (cutoff: 3 KDa) and centrifuged at 12,000 r/min for 1 h at 4 °C; 1 mL of the supernatant was collected and transferred to a 10 mL volumetric flask. The supernatant was diluted with methanol to 10 mL, followed by ultrasonic dispersion for 30 min and filtering through a 0.22 μm microporous filter membrane. The amount of free API in the PLGA-API-NPs (*W_f_*) was calculated using HPLC. In detail, an API standard solution (400 μg/mL in methanol) was prepared by dissolving 10 mg API in 25 mL methanol and storing the solution at 4 °C for later use. The API mixture concentration after demulsification with methanol was measured by HPLC at 210 nm using an Agilent 1100 system with a UV detector and an auto-sampler device. Separation was performed on an analytical column (Welchrom-C18, 5 μm, 4.6 × 150 mm) with a guard column at room temperature. The mobile phase was 41% acetonitrile in water solution (*v*/*v*) at a flow rate of 1 mL /min. Calibration curves were constructed and the volume injected into the HPLC system was 20 μL. There were linear relationships from 1 to 40 μg/mL (Y = 76.302X − 2.9938, *R*^2^ =0.9997). The encapsulation efficiency (EE) and DL were calculated according to the following formula:

$$EE\;(\%)=\frac{\left(W_{t}-W_{f}\right)}{W_{t}}\times 100\%$$

$$DL\;(\%)=\frac{(Wt-Wf)\;}{Wn}\times 100\%$$

where *W_t_* is the total amount of API (mg), *W_f_* is the free amount of API (mg), and *W_n_* is the weight of the PLGA-API-NPs (mg).

### 3.4. API Release from HA-Coated PLGA-API-NPs

A 1 mL amount of each of free API, PLGA-API-NPs, and HA-PLGA-API-NPs solutions (matched same API concentrations) were respectively added to three treated dialysis bags, which were clamped and placed in different sealed beakers with 10 mL of release media. Due to the low solubility of the API in water but good solubility in ethanol, the release medium was composed of PBS and ethanol in a ratio of PBS (pH 7.4) to ethanol of 4:1. The release medium was mixed using as shaking incubator (37 °C, 100 rpm). At each predetermined timepoint, 1 mL of the release medium was removed, filtered through the polycarbonate ester membrane (0.22 μm), and replenished to the container with the same amount of equal temperature medium. The API concentration was measured using HPLC, and the cumulative release percentage were calculated according to Formula (II):

$$Q\left(\%\right)=\frac{\sum_{n=i}^{i-1}C_{i}V_{i}+C_{t}V}{W}\times 100\%$$

where *C_i_* and *C_t_* are API concentrations before and at time t, respectively, *V_i_* is the sampling volume (mL), *V* is the total volume of released medium (mL), and *W* is the drug loading of the PLGA-NPs (μg).

### 3.5. In Vitro Cytotoxicity Studies

The anticancer effects of the HA-PLGA-API-NPs were assessed via MTT assay according to the manufacturer’s protocol. HRT-18 and HT-29 cells were treated with free API, PLGA-API-NPs, CS-PLGA-API-NPs, HA-PLGA-API-NPs, or blank HA-PLGA-NPs (as a control) for 24 h. The data represented the percentage of surviving cells, calculated as the mean values from 6 replicates.

### 3.6. In Vitro Cellular Uptake Assay

The cellular uptake of HA-PLGA-NPs was assessed by laser confocal microscope (Nikon A1, Japan) and flow cytometry using Dio-labeled HA-PLGA-API-NPs in vitro. Briefly, HRT-18 and HT-29 cells were seeded in 6-well plates (2 × 10^6^ cells/well), incubated overnight in CO_2_ incubator at 37 °C, then treated with 2 mL of fresh cell medium containing PLGA-DiO-NPs or HA-PLGA-DiO-NPs for 4 h in a CO_2_ incubator at 37 °C. The final concentration of DiO was 2 μg/mL in all groups. The final concentration of HA-PLGA-API-NPs was 0.1 mg/mL, and the equivalent total concentration of apigenin was 3 µg/mL. To explore the role of HA in CD44-mediated cellular uptake, the cells were pre-treated with HA (5 mg/mL) for 0.5 h before being incubated with DiO-labeled HA-PLGA-NPs. The cell nuclei were stained with DAPI and observed using a fluorescence microscope. Cell samples were collected, washed, and resuspended in PBS for flow cytometry analysis.

### 3.7. Establishment of an Animal Solid Tumor Model

Animal experiments were approved by the Institution Animal Care and Use Committee (No. HSDYXY-2022040). Healthy female BALB/C nude mice (4 weeks old) were supplied by Weitong Lihua Laboratory Animal Technology Co., Ltd. (Beijing, China). HRT-18 or HT-29 cells (1.5 × 10^7^ in 0.1 mL PBS) were injected subcutaneously into the left anterior armpit of the mice. The mice were subsequently monitored daily for general health and tumor progression. Once palpable tumors developed, details including the size and volume of the tumors were documented regularly.

### 3.8. In Vivo Near-Infrared Fluorescence (NIRF) Imaging

The mice bearing HRT-18 or HT-29 tumors (reached ~150 mm^3^) were randomly divided into four groups (*n* = 6) and injected through the tail vein with DiR-labeled PLGA-NPs or DiR-labeled HA-PLGA-NPs. At each predetermined timepoint, anesthetized mice were imaged via in vivo fluorescence imaging. At the end of the study, those mice were dissected and the main organs and tumors were collected for fluorescence intensity quantification.

### 3.9. Statistical Analysis

All data are presented as the mean ± standard deviation (SD). Following variance homogeneity testing, the differences between the groups were analyzed by one-way ANOVA. A *p*-value < 0.05 was deemed to indicate statistical significance.

## 4. Discussion

Despite promising antitumor properties, the poor water solubility and swift metabolic degradation of API hamper its delivery and efficacy [25]. To address these limitations, various drug delivery systems have been explored to improve the therapeutic effectiveness of API. Among these, PLGA NPs stand out as effective carriers due to their biocompatibility, biodegradability, small size, particulate structure, and absence of toxic metabolites [11]. However, unmodified PLGA NPs are primarily taken up by organs with significant blood flow, limiting absorption by colon cancer cells and resulting in severe side effects [26]. To enhance the cell uptake by colon cancer cells, particularly those expressing high levels of CD44, a receptor-selective drug delivery system (HA-PLGA-API-NPs) was successfully developed in this study; the molecules can selectively bind to the CD44 receptors on the surface of colon cancer cells and facilitate the targeted delivery of the hydrophobic anti-tumor drug API to colon cancer cells with high CD44 expression.

Recent investigations into receptor-selective drug delivery systems have demonstrated that targeting ligands can enhance the entry of nanoparticles into the target tissues through ligand–receptor binding, while safeguarding the active drug payload against non-specific delivery [27,28]. The targeting ligands enable nanoparticles to bind to cell surface receptors and enter cells through receptor-mediated endocytosis [29,30]. In our study, we sequentially coated the surface of PLGA-API-NPs with CS and HA. This was achieved through the electrostatic adsorption between CS and PLGA, followed by electrostatic adsorption with HA. This characteristic facilitated the specific binding of HA-PLGA-API-NPs to colon cancer cells expressing CD44 receptors, enhancing the internalization of HA-PLGA-API-NPs and improving the bioavailability of API.

The newly prepared HA-PLGA-API-NPs possessed a mean particle size of 254 ± 6.23 nm, a DL of 3.19 ± 0.03%, and an EE of 90.56 ± 0.57%, indicating excellent reproducibility. The stability of HA-PLGA-API-NPs was thoroughly evaluated; they showed satisfactory stability over 5 days with no significant changes in particle size and drug loading. It is worth noting that lyophilization may offer further improvements in nanoparticle stability in future studies, which is crucial for clinical applications. The PLGA-API-NPs exhibited a rapid initial release rate, primarily as a consequence of the rapid degradation of PLGA in the release solution with a lactate/glycolic acid ratio of 75:25, resulting in the release of most of the encapsulated API within 12 h. In contrast, HA-PLGA-API-NPs displayed a slower initial release, followed by sustained release after 12 h. This behavior could be attributed to the presence of the outer HA and CS coatings, which effectively delayed drug release [31]. Neither nanoparticles achieved a cumulative release rate of 80% within 48 h, suggesting a fraction of the API remains tightly entrapped in the PLGA matrix for gradual release over time [32]. Future studies are needed to characterize the prolonged in vitro release kinetics.

MTT assays were utilized to assess the cytotoxicity of HA-PLGA-API-NPs, and flow cytometry was used to analyze cellular uptake. HA-PLGA-API-NPs showed strong targeting to CD44-overexpressing HT-29 cells and had minimal cytotoxicity towards HRT-18 cells but higher cytotoxicity towards HT-29 cells. This suggested that HA modification significantly enhanced the targeting selectivity of NPs for cells with high CD44 expression. The flow cytometry results also showed the highest fluorescence intensity in HT-29 cells after HA-PLGA-DiO-NPs treatment. Fluorescence imaging clearly demonstrated greater uptake of HA-PLGA-DiO-NPs in CD44-high HT-29 cells, confirming the effective HA targeting of colon cancer cells. Consequently, ligand modifications can alter the nanoparticle–receptor interactions, influencing the in vitro and in vivo behaviors of the nanoparticulate system [33]. Nonetheless, additional research is warranted to explore the impact of varying molecular weights and densities of HA on PLGA nanoparticles, aiming for a comprehensive understanding of their effects on cytotoxicity and cellular uptake. Such investigations will establish a solid theoretical foundation for the development of targeted therapy.

Small animal in vivo fluorescence imaging techniques were employed to monitor the nanoparticle distribution in tumor-bearing mice. HA-PLGA-API-NPs exhibited significant targeting in high-CD44-expressing colon cancer mice models, whereas PLGA-API-NPs lacked specificity. The fluorescence intensity of PLGA-DiR-NPs peaked at 4 h and then declined, possibly due to the rapid initial DiR release followed by a slower release and metabolic processes. However, the fluorescence intensity of HA-PLGA-DiR-NPs continuously increased, reaching its maximum at 8 h. The delayed DiR release can be attributed to the HA modification resulting in a more controlled release, which was consistent with the in vitro results. The significant accumulation of nanoparticles observed in the livers of the mice potentially stemmed from the hepatic uptake of larger nanoparticles [34]. Subsequent investigations on this subject should focus on devising methods to decrease PLGA nanoparticle size without significantly compromising the drug-loading capacity, which may minimize nanoparticle uptake and improve the accumulation of the drug at the liver tumor sites.

Overall, the HA-PLGA-API-NPs developed in this study offered several significant advantages. Firstly, HA-PLGA-API-NPs achieved a more targeted delivery of the drug API to colon cancer tissues overexpressing CD44 receptors than traditional PLGA nanoparticles did. In contrast to HA-coated PLGA NPs without CS, the addition of CS further promoted the binding of HA with PLGA through electrostatic adsorption. This enhanced the stability of the drug during transportation and storage, reducing the risk of degradation. In summary, HA-PLGA-API-NPs represent a promising solution for addressing the challenges of drug delivery in colon cancer therapy, offering targeted and stable delivery for improved treatment outcomes.

## 5. Conclusions

In this study, we developed HA-PLGA-API-NPs to enhance the stability of API and enable the targeted delivery of the drug specifically to colon cancer tumors with high CD44 expression. HA-PLGA-API-NPs were uniform in size, with good dispersibility and stability via particle size and zeta potential measurements. Drug release assays confirmed that HA-PLGA-API-NPs had a stronger sustained-release capability than PLGA-API-NPs after 12 h. The cellular uptake of HA-PLGA-API-NPs was significantly enhanced in HT-29 cells compared with HRT-18 cells due to their specificity towards the CD44 receptors on the membrane of HT-29 colon cancer cells. In vivo, HA-PLGA-DiR-NPs showed enhanced targeting specificity towards the HT-29 ectopic tumor model in nude mice in comparison with PLGA-DiR-NPs. These results confirmed that HA-PLGA-API-NPs were an effective drug delivery system for API that elevated the therapeutic concentration of API at the tumor site, exhibited excellent specificity, and minimized concerns about off-target effects. This approach holds extensive potential for selectively targeting and effectively treating chemotherapy-resistant tumor cells.

## Figures and Tables

**Table 1:** Stability of HA-PLGA-API-NPs at 4 °C and 25 °C (*n* = 3).

Temperature (°C)	Time (d)	Particle Size (nm)	Drug Loading (%)
	0	245 ± 4.55	3.27 ± 0.04
4	5	255 ± 3.25	3.06 ± 0.05
	10	249 ± 5.67	2.89 ± 0.05
	0	245 ± 3.22	3.27 ± 0.06
25	5	258 ± 2.87	2.99 ± 0.06
	10	267 ± 4.55	2.66 ± 0.05

## Data Availability

The data presented in this study are available on request from the corresponding author. The data are not publicly available due to restrictions of privacy.
